# Shared Ancestry and Signatures of Recent Selection in Gotland Sheep

**DOI:** 10.3390/genes12030433

**Published:** 2021-03-17

**Authors:** Seyed Mohammad Ghoreishifar, Christina Marie Rochus, Sima Moghaddaszadeh-Ahrabi, Pourya Davoudi, Siavash Salek Ardestani, Natalia A. Zinovieva, Tatiana E. Deniskova, Anna M. Johansson

**Affiliations:** 1 Department of Animal Science, University College of Agriculture and Natural Resources, University of Tehran, Karaj 31587-11167, Iran; m.goreishi@ut.ac.ir; 2 Animal Breeding and Genomics, Wageningen University and Research, P.O. Box 338, 6700 AH Wageningen, The Netherlands; christina.rochus@gmail.com; 3 Department of Animal Science, Faculty of Agriculture and Natural Resources, Islamic Azad University, Tabriz Branch, Tabriz 5157944533, Iran; s.moghaddaszadeh@iaut.ac.ir; 4 Department of Animal Science and Aquaculture, Dalhousie University, Truro, NS B2N5E3, Canada; pourya.davoudi@dal.ca (P.D.); siasia6650@gmail.com (S.S.A.); 5 L.K. Ernst Federal Research Center for Animal Husbandry, 142132 Podolsk, Russia; n_zinovieva@mail.ru (N.A.Z.); horarka@yandex.ru (T.E.D.); 6 Department of Animal Breeding and Genetics, Swedish University of Agricultural Sciences, SE-75007 Uppsala, Sweden

**Keywords:** Gotland sheep, population structure, pelt quality, selection signatures, BayeScan

## Abstract

Gotland sheep, a breed native to Gotland, Sweden (an island in the Baltic Sea), split from the Gute sheep breed approximately 100 years ago, and since, has probably been crossed with other breeds. This breed has recently gained popularity, due to its pelt quality. This study estimates the shared ancestors and identifies recent selection signatures in Gotland sheep using 600 K single nucleotide polymorphism (SNP) genotype data. Admixture analysis shows that the Gotland sheep is a distinct breed, but also has shared ancestral genomic components with Gute (~50%), Karakul (~30%), Romanov (~20%), and Fjällnäs (~10%) sheep breeds. Two complementary methods were applied to detect selection signatures: A Bayesian population differentiation F_ST_ and an integrated haplotype homozygosity score (iHS). Our results find that seven significant SNPs (*q*-value < 0.05) using the F_ST_ analysis and 55 significant SNPs (*p*-value < 0.0001) using the iHS analysis. Of the candidate genes that contain significant markers, or are in proximity to them, we identify several belongings to the keratin genes, *RXFP2*, *ADCY1*, *ENOX1*, *USF2*, *COX7A1*, *ARHGAP28*, *CRYBB2*, *CAPNS1*, *FMO3*, and *GREB1*. These genes are involved in wool quality, polled and horned phenotypes, fertility, twining rate, meat quality, and growth traits. In summary, our results provide shared founders of Gotland sheep and insight into genomic regions maintained under selection after the breed was formed. These results contribute to the detection of candidate genes and QTLs underlying economic traits in sheep.

## 1. Introduction

Gotland sheep are a Swedish breed prized for their pelts that are uniformly grey in color with curly fleece. Gotland sheep are native to Gotland, a Swedish island in the Baltic Sea [1,2]. They belong to the North European short-tailed sheep type, sheep breeds that are characterized in part by their short tails and are found in Northern Europe from Russia to Iceland [2]. Gotland sheep separated over a hundred years ago from Gute sheep, a local Swedish breed [2]. Gotland sheep are raised commercially, have a relatively higher effective population size, and lower inbreeding compared to other Swedish North European short-tailed sheep breeds [1]. This can be attributed to historic crossbreeding. The Gotland sheep breed became very different from the Gute sheep breed in only a few decades, and it is likely that some crossbreeding with other breeds occurred to improve some traits. Although there is no clear evidence of which breeds were introgressed, Romanov sheep and Karakul sheep have been mentioned as ancestors in texts for the public about the history of Gotland sheep [3]. At least experimental crosses between Gotland sheep and Karakul sheep have occurred [4]. Also, the striking phenotypic resemblance between the Gotland sheep (Figure S1) and the Romanov sheep, where they share the black head and legs and grey wool, and the curl of the pelt of Karakul sheep makes those two breeds interesting to compare with. The Romanov sheep breed is native to Russia and are also North European short tail sheep. This breed is well-known for its reproductive performance and is raised in many countries for crossbreeding with local breeds [5]. In contrast to Gotland, Gute, and Romanov sheep, Karakul sheep is a fat-tailed sheep breed [6]. Fat-tailed sheep were originally a desert animal that stored fat in its tail to be mobilized during periods of food scarcity [6]. The Karakul sheep breed is an ancient breed that is now commonly raised in both Asia and Africa [6]. Pelts of Karakul lambs are historically referred to as “Persian lamb”, and these sheep are also sources of milk, meat, and fiber [6].

Breeding efforts in Gute sheep have been focused on conservation rather than genetic improvement of production and marketable traits [7]. However, in Gotland sheep, selection to improve pelt quality, such as the curl of the fleece, the consistent color of the pelt, and thickness of fleece, started after the breed was formed in about 1930, and continues to be the focus in the current breeding program [2]. Hence, along with natural selection, artificial selection shapes the genetic architecture of breeds leaving signatures in the genome that might be detectable.

With cost-effective genotyping technologies, it is possible to genotype several individuals from different breeds to scan their genome to uncover regions that are under putative selection [8]. The idea behind selection signature detection theory is that the frequency of alleles that are under selection can vary in opposite directions (low or high), resulting in stretches of consecutive homozygous genotypes, or in modifying the length and frequency of haplotypes around the region [9]. When selection signatures are identified, they can help us understand the processes that cause diversity among breeds, and locate candidate genes involved in a phenotype of interest. Other studies of selection signatures in sheep [10,11,12,13,14,15] and other animal species [8,16,17,18,19,20,21,22] have illustrated how these methods can find genomic regions potentially related to economic or adaptation-related traits. Thus, identification of recent selection signatures in Gotland sheep will help detect novel genomic regions associated with economically important traits, such as pelt and fleece quality.

The purpose of our study was to estimate population structure and identify candidate genes underlying recent artificial selection in Gotland sheep. This study builds on other studies of population structure in Swedish North European short-tailed sheep breeds [1] by including genomic data from two additional breeds, Romanov [13,14] and Karakul sheep [13], that were likely used in the past to crossbreed with Gotland sheep. The results of selection signatures analyses in this study could contribute to genomic predictions through weighting genomic relationship matrix strategies based on selection signature values [23,24] in Gotland sheep, and be beneficial for optimizing the single nucleotide polymorphism (SNP) panels that are widely applied in sheep genomic breeding programs.

## 2. Materials and Methods

### 2.1. Samples and Data Quality Control

High-density genotype data (Ovine HD 600K SNP array) of Swedish sheep breeds [1] was downloaded from DRYAD: https://doi.org/10.5061/dryad.34tmpg4gj (accessed on 11 March 2021). This dataset included SNP genotypes from five Swedish sheep breeds, of which only Gotland (*n* = 19), Gute (*n* = 22), and Fjällnäs (*n* = 10) sheep were retained for the subsequent analyses (Table 1). To study the population structure of Gotland sheep, samples of Romanov (*n* = 24) and Karakul (*n* = 20) sheep from Russia [13] and Romanov sheep (*n* = 10) were sampled from French commercial farms [14] were also included.

First, SNPs that were duplicated in the map file (i.e., identified using duplicated function in R [25]) and didn’t have an identified position in the sheep genome assembly [26] were removed from each dataset using the --exclude the option of the PLINK v1.9 [27] program. Next, we merged the three datasets, including 502,008 common SNPs, into a single PLINK v1.9 [27] binary format. Data quality was checked using PLINK v1.9 [27] for a total of 105 individuals. All genotyped individuals had a sample call rate greater than 0.90 and were included in this study. Finally, SNPs with a call rate less than 0.95 (*n* = 6871) and a minor allele frequency (MAF) less than 0.05 (*n* = 30,525) were discarded.

### 2.2. Population Genetic Structure Analysis

Two main approaches were adopted to study population structure and to identify and date admixture events in the Gotland sheep genome including (1) a Principal Component (PC) analysis and (2) a method to estimate individual ancestry coefficients.

#### 2.2.1. Principal Component (PC) Analysis

We used the --ibs-matrix command in PLINK v1.9 [27] to estimate an identical by state (IBS) distance matrix among the individuals included in our dataset. The IBS matrix, was used to calculate PCs, with the prcomp R [25] function. Next, the first two PCs were plotted to visualize population structure using R [25].

#### 2.2.2. Individual Ancestry Coefficients

Individual ancestry coefficient analysis, which is based on sparse non-negative matrix factorization algorithms, was performed using sNMF v1.2 [28] software from the LEA R package [29] (using the snmf function). To this end, binary files were converted to “.ped” and “.map” formats using the PLINK v1.9 [27] --recode function. Once generated, “.ped” and “.map” files were transformed into the “.geno” format required as input by sNMF. For this format conversion, we used the “ped2geno” program implemented in the command-line version of the sNMF v1.2 [28] software. Individual ancestry coefficients were calculated using the snmf function from the LEA package [29] with K (i.e., number of hypothetical ancestors) values ranging from 2 to 10 and with five iterations each. The optimal number of ancestors had the lowest cross-entropy criterion. Finally, the individual ancestry coefficients (from K = 2 to the optimal number of K) were plotted using R [25].

### 2.3. Identifying Genomic Regions Showing Recent Selection Signatures

We used two different methods to identify selection signatures in the genome of Gotland sheep. These methods were Bayesian population differentiation (F_ST_) [30] and integrated haplotype homozygosity score (iHS) [9].

#### 2.3.1. Bayesian Population Differentiation (F_ST_)

High-density genotype data, 464,612 SNPs, was used to identify significant differences in allele frequencies between Gotland and Gute sheep breeds. Bayesian population differentiation F_ST_ implemented in the BayeScan program [30] was used to detect regions under selection, i.e., loci that have been subjected to selection and show significantly higher values of F_ST_ than those expected under neutrality. SNP genotype data from Gotland and Gute sheep were isolated using the PLINK v1.9 [27] --keep function, and then converted to the format required by the BayeScan program [30] using a custom R [25] script. All parameters for running the BayeScan program [30], except for the prior odds of 1000 (-pr_odds 1000), were set as default, including 20 pilot runs with 5000 iterations (-nbp 20 -pilot 5000), a burn-in of 50,000 iterations (-burn 50,000), and a thinning interval of 10 with 5000 iterations (-n 5000 -thin 10) resulting in a total number of 100,000 iterations. We also discarded those SNPs that were monomorphic in both populations (*n* = 17,399) using the -d option in BayeScan [30]. This option accepts a vector file, including the order of each SNP that should be excluded. Finally, to control the number of false positives in our multiple testing analysis (including the null hypothesis testing for 447,213 markers), SNPs with a *q*-value < 0.05 were statistically significant.

#### 2.3.2. Integrated Haplotype Homozygosity Score (iHS)

To calculate iHS [9], un-phased SNP genotypes needed to be converted to phased haplotypes. We used SHAPEIT v2 [31] for haplotype phasing of the autosomal genome. To gain phasing accuracy, we included all the breeds used in this study. The SHAPEIT2 [31] parameters conditioning states of 400 (--states 400) and effective population size of 500 (--effective-size 500) were used. A high resolution ovine genetic map [32] was applied to accompany SHAPEIT2 [31] for haplotype phasing to correct for the variation in recombination rate along the ovine genome. To calculate the iHS statistics, the phased haplotypes of Gotland sheep were extracted. For SNPs with a MAF > 0.05, the ancestral/derived alleles were randomly assigned, as reported in a previous study [20]. The selscan program [33] was run to calculate the iHS with the default parameters, including the scale parameter of 20,000, a max gap of 200,000, and EHH (Extended Haplotype Homozygosity) cutoff value of 0.05. The iHS results for different chromosomes were combined in a single file and then frequency normalized in 100 bins using the norm package that accompanies selscan program [33]. Two-sided *p*-values were calculated using *p*_iHS_ = [1−2|Φ(iHS)−0.5|]. In this equation Φ(iHS) denotes the Gaussian cumulative distribution function, which was calculated using the pnorm R [25] function, and *p*_iHS_ was the two-sided *p*-value for testing the null hypothesis (i.e., no selection). A *p*_iHS_ value less than 0.0001 was significant.

#### 2.3.3. Identification of Genes Located in the Regions Showing Selection Signature

To identify candidate genes located in genomic regions under selection, we used ovine gene annotation data (Ovis-aries.OAR_v3.1.100) downloaded from the Ensembl genome browser website: ftp://ftp.ensembl.org/pub/release-100/gtf/ovis_aries/ (accessed 12 March 2021) [26,34]. Genomic regions were 400 Kb window genomic region from 200 Kb upstream to 200 Kb downstream of significant SNP marker identified by the Bayesian F_ST_ (*q*-value < 0.05) or iHS (*p*_iHS_ < 0.0001). Finally, we performed a literature review to annotate the functions of the identified genes.

## 3. Results and Discussion

### 3.1. Principal Component (PC) Analysis

**Figure 1:**
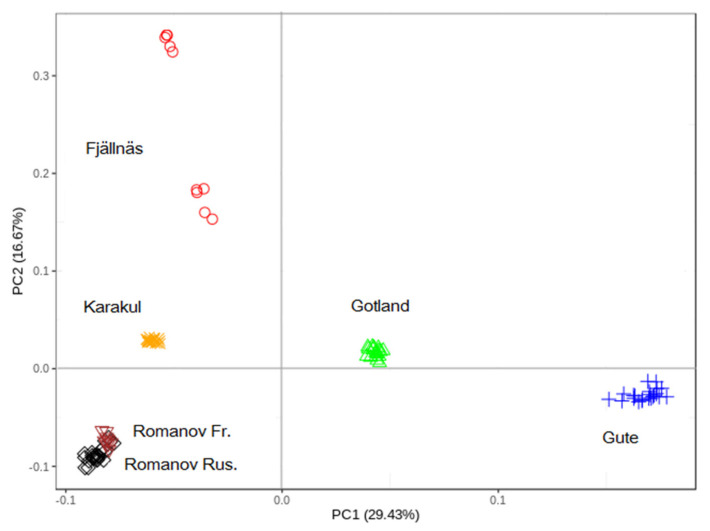
Principal Component (PC) analysis of Gotland, Gute, Fjällnäs, Romanov, and Karakul sheep breeds.

A total number of 464,612 autosomal SNP genotypes from 105 animals (of which 19 individuals were Gotland sheep) with a mean genotyping call rate of 99.39% remained after the quality control process. The average distance (±SD) between adjacent SNPs was 5.25 (± 5.74) Kb. As presented in Figure 1, the first two PCs, which explained 46.1% of genetic diversity among the five breeds, showed that all breeds are in different clusters, and individuals within each breed clustered close together. The first two PCs illustrated the intermediate position of Gotland sheep individuals in relation to the other breeds, i.e., Gute on one side, and Karakul, Romanov, and Fjällnäs sheep on the other side. These results suggest that the Gotland breed has some shared ancestry (or genetic relationship) with Karakul, Romanov, and Fjällnäs sheep in addition to Gute sheep.

### 3.2. Population Admixture

**Figure 2:**
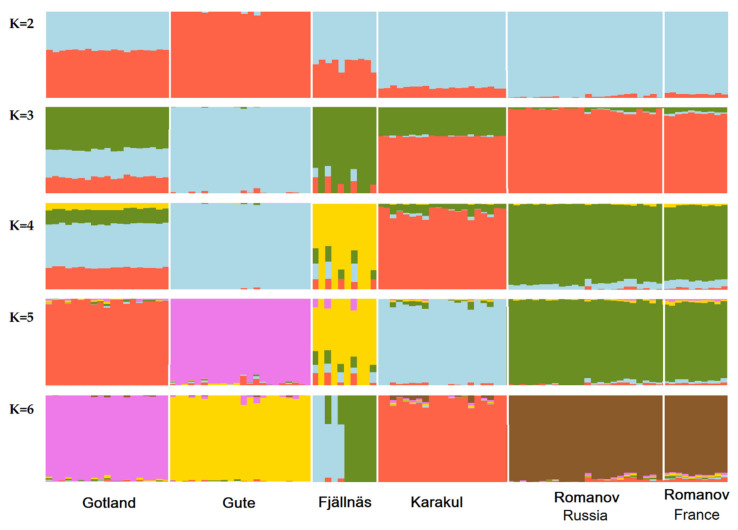
Population structure (shared ancestry) of Gotland, Gute, Fjällnäs, Romanov, and Karakul sheep breeds and a different number of ancestral populations (i.e., K = 2, 3, 4, 5, 6). K = 6 was the optimal number of ancestral populations because it had the lowest cross-entropy criterion.

The population structure of Swedish indigenous sheep breeds, including Gotland and Gute sheep, has already been studied with high-density SNP genotype data [1]. In the current work, however, we focused on Gotland sheep with the inclusion of data from previous studies on Russian [13] and French [14] sheep. The results of the PC analysis and analyses from the literature [1] are consistent with Gute, Romanov, and Karakul being related to Gotland sheep. Thus, an admixture analysis was conducted to identify shared ancestry in Gotland sheep with different numbers of clusters (K), an illustration of the hypothetical number of ancestral populations (Figure 2). Individual ancestry coefficients were estimated assuming 2 to 10 clusters (K), of which K = 6 was the optimal K obtained based on the cross-entropy criterion. Assuming two ancestral populations, Gute sheep was the most distinct breed, followed by Romanov, Karakul, and Fjällnäs. In K = 2, Gotland sheep had 45% shared ancestry with Romanov and Karakul sheep, and 55% shared ancestry with Gute sheep. Assuming K = 3, Gute sheep was the most distinct breed, followed by Romanov, Fjällnäs, and Karakul sheep. When the number of ancestral populations was assumed to be 4, Gotland sheep had ~50% shared ancestry with Gute sheep, ~30% with Karakul sheep, ~20% with Romanov sheep, and ~10% with Fjällnäs sheep. Assuming K = 5, all the breeds were distinct, while for K = 6, which was the optimal number of ancestral populations, Fjällnäs sheep were divided into two sub-clusters as was reported in a previous study that used the same Fjällnäs sheep genotype data [1]. The results support that Gotland sheep and Gute sheep have a common ancestry, but also show clear genetic similarities of Gotland sheep with the Romanov sheep and Karakul sheep, indicating a shared ancestry. We cannot find evidence in the literature about recent introgression of Romanov and Karakul sheep into Gotland sheep, but at least gene flow in the past between Romanov and Karakul sheep on Gotland is plausible because Gotland is an island in the Baltic Sea and not very far away from Russia by boat. The fact that Gute sheep is in its own cluster already at K = 2 is probably due to genetic drift because Gute sheep went through a bottleneck with very few individuals in the middle of the 20th century when it was about to become extinct and only had five adult rams in 1944 according to [35]. Thus, although some phenotypes were similar to the old type of sheep on Gotland, the Gute sheep does not represent the whole genetic diversity that was present in the ancestors of Gute sheep and Gotland sheep, only a small part of it. 

### 3.3. Identification of Selection Signatures with BayeScan and iHS

**Figure 3:**
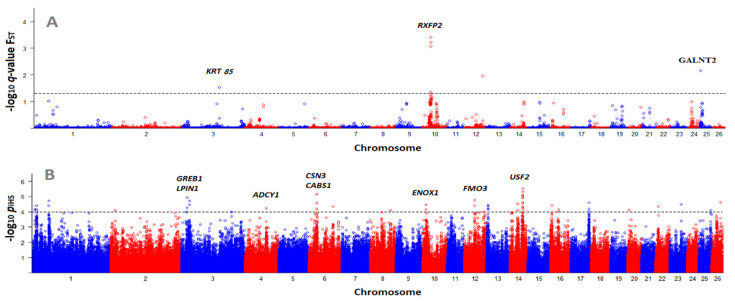
Manhattan plots for selective sweep analyses of Gotland sheep with the (**A**) Bayesian fixation index (F_ST_) algorithm, and (**B**) integrated haplotype homozygosity score (iHS) method. The dashed lines represent the threshold level for significance at *q*-value < 0.05 for F_ST_ and *p*_iHS_ < 0.0001 for iHS.

According to Figure 3A, we identified seven significant SNPs (in 4 different regions) using the BayeScan F_ST_ [30] between Gotland and Gute sheep breeds. The seven significant SNPs (F_ST_ > 0.4659) were 0.0016% of all polymorphic SNPs tested. The most significant SNP was located on OAR10 at 29,455,959 bp (Table 2). The genomic region (200 Kb upstream and downstream) of this significant SNP contained *RXFP2*, a gene under strong selection in other sheep breeds and associated with horned and polled phenotypes [11]. A study of whole genome sequencing data from Chinese sheep showed the correlation of *RXFP2* with horn length and shape [36]. Given the history of Gute and Gotland sheep, this result is expected. Part of the efforts to improve the Gotland sheep breed for commercial purposes was to select polled animals, while both sexes of Gute sheep are horned. This result shows the F_ST_ statistic can identify genes/alleles that are fixed in a population, even if the divergent selection has taken place relatively recently (in this example, approximately 100 years ago since [35] reported that in the years 1890–1910, most of the rams on Gotland had horns and also ewes often had horns).

We also found a significant SNP (*q*-value = 0.007) on OAR25 at 1,759,489 Kb, which was located inside the *PGBD5* gene. No association between *PGBD5* and a specific trait in livestock has been reported so far. However, in the vicinity of this SNP (i.e., 200 Kb upstream and downstream), we identified another candidate gene, *GALNT2*. An association between a polymorphism in *GALNT2* and serum lipid level was previously reported in humans [38]. This gene was also reported as a candidate gene associated with average daily gain (ADG) in Simmental beef cattle [39]. On OAR12 a SNP at 63,873,649 kb was significant (*q*-value = 0.011), which was located inside *EDEM3*. In sheep, *EDEM3* has been identified as a candidate gene for resistance to gastrointestinal nematodes [37]. This candidate gene highlights a challenge of studies that detect selective sweeps: Without phenotypes, we can’t know what trait has been selected on. In this case, *EDEM3* has been associated with three different phenotypes in other livestock species, and it is difficult to make an educated guess as to why this region has been under selection in Gotland sheep.

The last significant SNP identified by BayeScan was located on OAR3 at 133,648,712 (*q*-value = 0.03). In the surrounding genomic region of this SNP (200 Kb upstream and downstream), we identified the following genes: *KRT4*, *KRT3*, *KRT77*, *KRT1*, *KRT73*, *KRT72*, *KRT74*, *KRT75*, *KRT82*, *KRT84*, and *KRT85*. The last three genes are known as ty including *KRT85* in secondary follicles, were reported to be in association with bulb deflection and follicle curvature [40], suggesting a role of *KRT85* in the determination of follicle and fiber morphology. Of the type II keratin genes, *KRT83* was already reported to be associated with wool traits [41]. Type II keratin genes seem to encode proteins that are assembled into keratin intermediate filaments in the wool fiber, suggesting their role in wool phenotype.

A study was published with expression data from skin tissue of a Chinese pelt sheep [42] in which the keratin genes identified to be differentially expressed were epithelial. Considering the majority of Keratin genes identified in our study were also epithelial, and also both Swedish Gotland and the Chinese Tan sheep [42] are pelt sheep; therefore, it could be that epithelial keratin is playing a role in the quality of the pelt, but not necessarily the fleece. While there haven’t been other association studies in sheep identifying the other keratin genes we’ve found, keratin is an important component of skin and hair/fleece and deserves some mention. 

In comparison to Gute sheep that are bred for conservation, Gotland sheep are bred for pelt quality traits, such as curl and color of the fiber. Therefore, the identification of type II keratin genes involved in wool and fleece quality was expected in this study. However, there needs to be more research done into what the role is of the keratin genes we’ve identified. Indeed, comparing Gotland and Gute sheep genomes is an ideal situation because we should only find signatures associated with artificial selection (and not natural selection because they are raised in similar environments). Therefore, the wool/fiber Keratin genes may provide insights into the differences between Gute and Gotland pelts, as well as the variation within Gotland individuals, which deserves further investigation with more samples, recorded phenotypes, and whole genome sequencing that can help identify causal variants with greater power and precision.

Figure 3B is a Manhattan plot of (−log_10_) *p*-values for iHS statistics. In cattle, more than 50,000 SNPs are recommended to accurately detect selection signatures using an EHH-based statistic [17] such as iHS [9], and in this study, we used about 400,000 evenly distributed SNPs across the sheep genome. The varying number of SNPs were reported as significant by iHS statistic in different studies, e.g., References [16,43]. This may be affected by several factors, such as population itself, sample size, significance level defined by the researcher, density of SNP chip used, etc. In the current study, a total number of 55 SNPs were identified as significant (*p*_iHS_ < 0.0001), which was 0.014% of all SNPs (385,079 SNPs) tested for the null hypothesis. One hundred and twenty-three protein-coding genes were identified in the 200 Kb upstream and downstream of the significant SNPs (Additional file 1); of these candidate genes, 11 had significant SNPs located within them, including *TFAP2E*, *LPIN1*, *ENOX1*, *SPTLC3*, *SIPA1L3*, *GRK3*, *MYO18B*, *HLA-DMB*, *LIPN*, *LAMA1*, and *C10orf71*. The most significant SNP was identified on OAR14, and in the surrounding region of this SNP (i.e., 107 Kb downstream), we found *USF2*, among other genes (Figure 3B; Table 3). In a study of a local Chinese sheep breed, Zhang et al. [44] hypothesized that *USF2* and *USF1* may contribute to differential expression of *BMP7*, resulting in an increase in prolificacy. We found several other candidate genes already reported as being involved in fertility and reproduction-related traits, including *ADCY1*, *COX7A1*, *TYROBP*, and *ARHGAP28* (Figure 3B; Table 3). *ADCY1* is located 61.4 Kb downstream of a significant SNP we identified (with −log_10_ *p*_iHS_ = 4.24) on OAR4. Through the production of cAMP, this gene is involved in oocyte meiotic arrest and resumption [44]. *ADCY1* was also reported as a candidate gene under selection in a high fecundity goat breed compared with a low fecundity dairy breed in a study detecting selection signatures [45].

Another candidate gene in our study is *COX7A1* which is 82.8 Kb upstream of a significant SNP (−log_10_ *p*_iHS_ = 4.95) on OAR14. This gene is involved in the oxidative phosphorylation pathway, and was significantly enriched as observed by Tang et al. [46] in a highly prolific sheep breed. Indeed, the potential of cytoplasmic transmembrane and ATP content is augmented by the oxidative phosphorylation pathway, which is necessary for follicular maturation [63]. The observed pattern (i.e., up-regulation of *COX7A*) in a highly prolific ewe, may, therefore, be explained by providing more energy supply to follicular development that would result in high prolificacy [46]. *ARHGAP28* is 13.6 Kb downstream of the next most significant SNP (−log_10_ *p*_iHS_ = 4.5) on OAR 23. In a GWA study regarding twinning rate in cattle, the most significant SNP was identified in the proximity of two genes, one of which was *ARHGAP28* [62]. These authors hypothesized that *ARHGAP28* may directly be involved in the ovulation process [62]. *ARHGAP28* was also identified as among the progesterone-regulated genes in humans and mice as it demonstrated a −3.1 fold change in gene expression in a case group (i.e., women treated with anti-progestin) in comparison with a control group [64]. Indeed, we expected to identify some genes related to reproduction and fertility because prolificacy is economically beneficial for commercial producers and was likely selected after crossing with Romanov sheep. Most reproduction- and fertility-related traits are quantitative and have moderate to low heritability; therefore, our results may provide new insights into the genomic regions under selection in Gotland sheep that may explain a considerable amount of genetic variation for these traits.

In our study, we wanted to detect selection signatures maintained in the genome of Gotland sheep, for which two different, but complementary methods including F_ST_ and iHS were applied. Conceptualized by Weir and Cockerham [65], F_ST_ has been broadly used to study population differentiation and selection signatures in different species. Indeed, F_ST_ measures differences in allele frequency between two populations in each locus, and those loci showing the highest differences are deemed as selection signatures. In the current study, we used a Bayesian approach implemented in BayeScan to detect F_ST_ outliers. The sample size in our study was relatively small (i.e., Gotland *n* = 19; Gute: *n* = 22); however, the BayeScan algorithm accounts for the uncertainty in allele frequencies of small sample sizes. This method can handle a very small sample size without introducing bias, but has reduced statistical power to detect selection signals.

The average F_ST_ values between Gute and Gotland sheep obtained with BayeScan was 0.158, suggesting a relatively high genetic differentiation. This can be attributed to the fact that the additional breeds used in synthesizing Gotland sheep were relatively highly differentiated, as shown in the PC analyses. In the case of comparing two relatively distinct populations, a locus with a relatively high F_ST_ value may not provide sufficient evidence for divergent selection than the same value, while comparing two closely related populations. Moreover, previous studies reported that the F_ST_ approach, in most cases, suffers from type I error (false positives) and bias [10,66]. However, one considerable advantage of the Bayesian F_ST_, i.e., the BayeScan algorithm, is that false discovery rate (FDR) is used to account for multiple testing, and posterior probabilities are calculated for each locus. In a study on the performance of F_ST_ outliers using simulation, Lotterhos and Whitlock [67] showed that using default parameters in BayeScan can increase false positives, while increasing the prior odds can improve the performance of BayeScan. They also showed that a large number of available neutral loci can generate empirical *p*-values, and therefore, improve the performance of BayeScan [67]. Previous studies used medium-density SNPs and default parameters to detect selection signatures with BayeScan [30]. In our study, however, we used high-density SNPs and increased the prior odds parameter by 1000 (i.e., the default setting was 10) to decrease the false discovery rate and increase the power of detection. Therefore, we expected a very low rate of false positives from our BayeScan results from the two differentiated populations (the seven significant SNPs identified with BayeScan are likely not false positives).

Despite the conservative nature of EHH-based methods, our iHS [9] analysis identified more significant markers than the F_ST_ method (i.e., 55 significant markers identified by iHS while only 7 by F_ST_). No SNPs were found to be significant for both methods, which could be explained because iHS is powerful in detecting ongoing selection signatures with an intermediate allele frequency; while F_ST_ is useful in detecting selection where the target allele frequency is approaching fixation. Although none of our selective sweeps were found with both methods, we identified candidate genes associated with economically important traits. 

## 4. Conclusions

This research investigated the population structure of Gotland sheep. We showed that while Gotland sheep is a distinct breed, it has a shared ancestry with Gute, Karakul, Romanov, and Fjällnäs breeds. Analysis of population differentiation showed a high level of genetic differentiation between Gute and Gotland sheep breeds. Although the Gotland breed originated from the same population of Gute, this research showed important genes, e.g., pelt quality and horned/polled phenotype that are involved in their differentiation. We also identified genes in significant genomic regions underlying fertility traits. We expected to identify some genes related to reproduction and fertility because prolificacy is economically beneficial for commercial producers and was likely selected after crossing with Romanov sheep. Given that interpreting the results inferred from signatures of selection is not straightforward, a supplementary genome-wide association study with a larger sample size, and the use of whole-genome sequence data, may be beneficial to validate the results and determine biological functions of selective sweeps.

## Figures and Tables

**Table 1:** Description of sheep breeds used in this study.

Breed	Sample	Characteristic	Cite
Gute	Sweden (*n* = 22)	Primitive breed native to the island of Gotland (Sweden), the horned phenotype in both rams and ewes, bred for conservation purposes, short tail	[1]
Gotland	Sweden (*n* = 19)	Native to the island of Gotland (Sweden), split from the Gute sheep breed, probably crossed with other breeds, including Karakul and Romanov, polled phenotype, bred for commercial purposes (e.g., pelt quality), the fleece is curly	[1]
Fjällnäs	Sweden (*n *= 10)	Accepted officially as a breed in 2011, from northern Sweden, very small effective population size, has shared ancestry with Gotland breed	[1]
Karakul	Russia (*n* = 20)	Horned and polled phenotypes, fat tail, breed raised for fleece and meat	[13]
Romanov	Russia (*n* = 24) France (*n* = 10)	Native breed from Russia, known for its high prolificacy, lambing of litters, early sexually maturing age, year-round breeding	[13,14]

**Table 2:** Candidate genes in a region 200 Kb upstream and downstream of significant (*q*-value < 0.05) single nucleotide polymorphisms (SNPs) obtained with the F_ST_ method when comparing Gotland and Gute sheep breeds.

SNP Position	*q*-Value	Gene	Gene Position	Trait	Reference
10:29,455,959	0.0008	*RXFP2*	within	Horned/polled phenotypes	[36]
12:63,873,649	0.0100	*EDEM3*	within	Gastrointestinal nematodes	[37]
25:1,759,489	0.0100	*GALNT2*	96.7 Kb up	Growth and carcass traits	[38,39]
3:133,648,712	0.0300	*KRT85*	-	Wool quality and quantity	[40]

**Table 3:** Candidate genes in a region 200 Kb upstream and downstream of significant (*P*_iHS_ > 4.00) single nucleotide polymorphisms (SNPs) obtained with the iHS in Gotland sheep.

SNP Position	*p*_iHS_^1^	Gene Name	Gene Position ^2^	Trait	Reference
14:45,845,449	4.95	*COX7A1*	82.8 Kb up	Prolificacy	[46]
3:20,370,238	4.28	*GREB1*	6.4 Kb up	Muscle growth	[47]
3:20,576,558	4.94	*LPIN1*	Within	Milk fat, growth, carcass	[48,49,50]
3:28,665,400	4.72	*APOB*	6.4 Kb up	Cold acclimation	[51]
4:76,392,859	4.24	*ADCY1*	61.4 Kb down	Fecundity	[45]
6:85,405,971	4.34	*CSN3*	89.1 Kb down	Litter size	[52,53,54]
10:14,096,224	4.47	*ENOX1*	Within	Litter size	[55]
12:36,960,517	4.41	*FMO3*	132.2 Kb down	Fat deposition	[56]
12:37,377,415	4.77	*MYOC*	112.1 Kb down	Spermatogenesis	[57]
14:45,201,813	4.61	*USF2*	107.9 Kb down	Litter size	[44]
14:45,845,449	4.95	*CAPNS1*	75.8 Kb up	Growth, meat quality	[58,59]
14:45,538,588	4.27	*TYROBP*	175 Kb up	Prolificacy	[60]
16:29,966,767	4.15	*MRPS30*	75.5 Kb up	Fatty acid profile	[61]
23:40,285,834	4.50	*ARHGAP28*	13.6 Kb down	Twinning rate	[62]

^1^ Transformed values (i.e., −log_10_ *p*_iHS_) are shown. ^2^ Down and up refer to candidate gene position downstream or upstream of the significant SNP, respectively, and within indicates that the significant SNP was located within the candidate gene).

## Data Availability

The SNP genotyping data of Swedish sheep breeds presented in this study are openly available in DRYAD repository at https://doi.org/10.5061/dryad.34tmpg4gj, accessed on 9 March 2021.

## Supplementary Materials

The following are available online at https://www.mdpi.com/2073-4425/12/3/433/s1, Figure S1: Swedish Gotland sheep, Additional file 1.
