# Supplementary figures and images for: Shared Ancestry and Signatures of Recent Selection in Gotland Sheep
Source: Genes (Basel). 2021 Mar 17;12(3):433. doi: 10.3390/genes12030433 (PMC8002741; doi:10.3390/genes12030433)

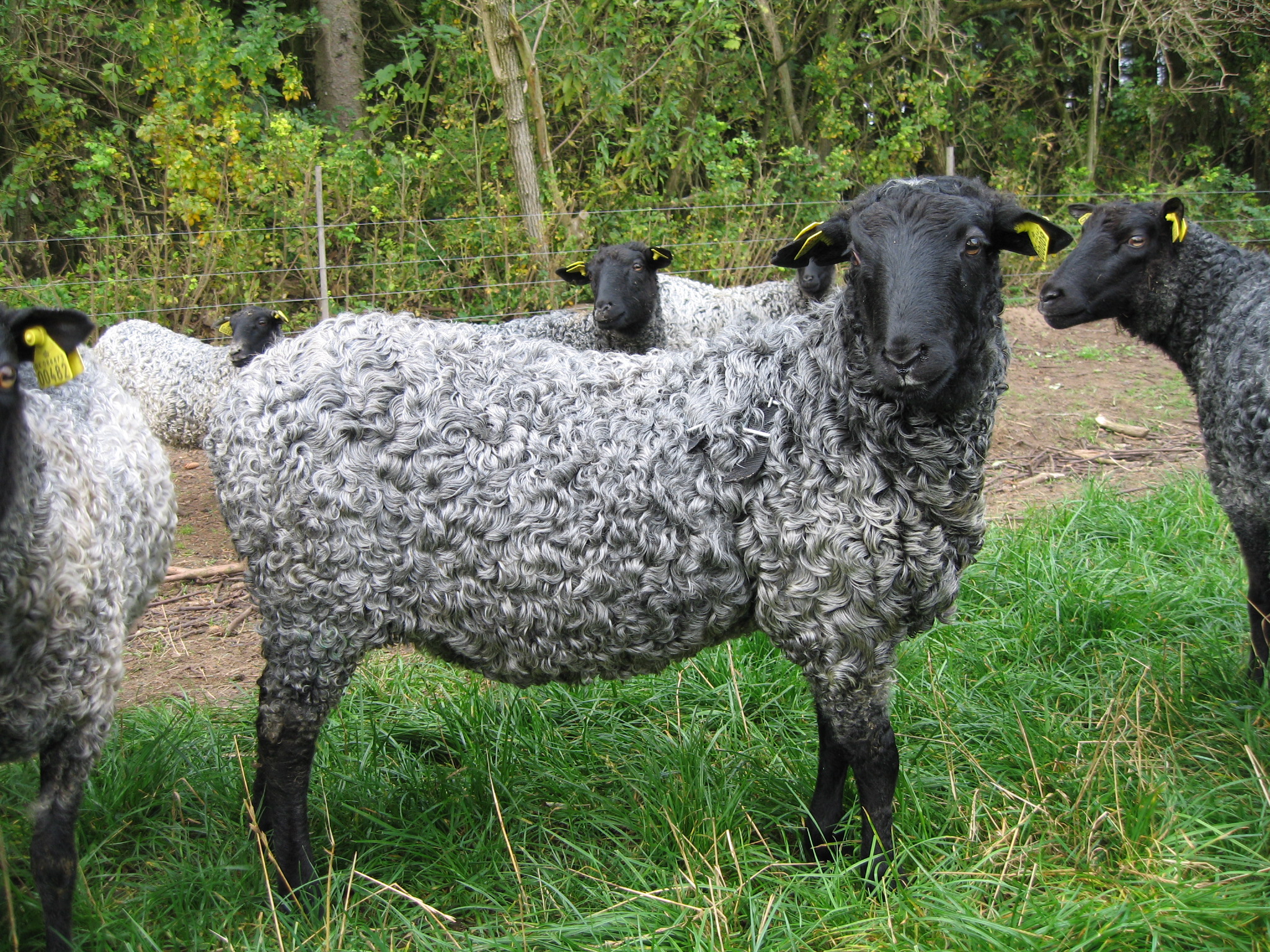

Supplement: Supplementary file 1 [file genes-12-00433-s001.zip › Figure S1.jpg]
